# Does bracing affect bone health in women with adolescent idiopathic scoliosis?

**DOI:** 10.1186/s13013-015-0031-1

**Published:** 2015-02-18

**Authors:** Nasreen Akseer, Kimberly Kish, W Alan Rigby, Matthew Greenway, Panagiota Klentrou, Philip M Wilson, Bareket Falk

**Affiliations:** Department of Kinesiology, Faculty of Applied Health Sciences, Brock University, 500 Glenridge Avenue, St. Catharines, ON L2S 3A1, Canada; Centre for Bone and Muscle Health, Faculty of Applied Health Sciences, Brock University, 500 Glenridge Avenue, St. Catharines, ON L2S 3A1, Canada; Behavioral Health Sciences Research Lab, Faculty of Applied Health Sciences, Brock University, St. Catharines, Canada; Michael G. DeGroote School of Medicine, Niagara Regional Campus, McMaster University, 500 Glenridge Avenue, St. Catharines, ON L2S 3A1, Canada

**Keywords:** Adolescence, Adulthood, Bone, Brace, DXA, Exercise, Female, Growth, Maturation, Nutrition, Physical activity

## Abstract

**Purpose:**

Adolescent idiopathic scoliosis (AIS) is often associated with low bone mineral content and density (BMC, BMD). Bracing, used to manage spine curvature, may interfere with the growth-related BMC accrual, resulting in reduced bone strength into adulthood. The purpose of this study was to assess the effects of brace treatment on BMC in adult women, diagnosed with AIS and braced in early adolescence.

**Methods:**

Participants included women with AIS who: (i) underwent brace treatment (AIS-B, n = 15, 25.6 ± 5.8 yrs), (ii) underwent no treatment (AIS, n = 15, 24.0 ± 4.0 yrs), and (iii) a healthy comparison group (CON, n = 19, 23.5 ± 3.8 yrs). BMC and body composition were assessed using dual-energy X-ray absorptiometry. Differences between groups were examined using a oneway ANOVA or ANCOVA, as appropriate.

**Results:**

AIS-B underwent brace treatment 27.9 ± 21.6 months, for 18.0 ± 5.4 h/d. Femoral neck BMC was lower (p = 0.06) in AIS-B (4.54 ± 0.10 g) compared with AIS (4.89 ± 0.61 g) and CON (5.07 ± 0.58 g). Controlling for lean body mass, calcium and vitamin D daily intake, and strenuous physical activity, femoral neck BMC was statistically different (p = 0.02) between groups. A similar pattern was observed at other lower extremity sites (p < 0.05), but not in the spine or upper extremities. BMC and BMD did not correlate with duration of brace treatment, duration of daily brace wear, or overall physical activity.

**Conclusion:**

Young women with AIS, especially those who were treated with a brace, have significantly lower BMC in their lower limbs compared to women without AIS. However, the lack of a relationship between brace treatment duration during adolescence and BMC during young adulthood, suggests that the brace treatment is not the likely mechanism of the low BMC.

## Background

Adolescent idiopathic scoliosis (AIS) is a persistent lateral curvature of the spine^1^. Its etiology and pathogenesis remain unknown. AIS is generally diagnosed between age 10 yrs and the age of skeletal maturity, up to age 18–20 years [1]. Its prevalence is 2-4% among children from 10 to 16 years and is higher in girls compared to boys [2].

Treatment of AIS depends on the Cobb’s angle and location of the curve, and the patient’s growth status. In general, for individuals with curves of 20-40°, a rigid brace is used if progression is documented and if the patient has substantial remaining growth [3]. The primary aim of bracing is to prevent further curve progression during the growing years [3,4]. A recent study by Weinstein and his colleagues have shown that bracing for AIS, significantly decreased the progression of high-risk curves to the threshold for surgery [5]. Most braces are rigid, immobilizing the trunk and limiting the use of core muscles [6,7]. In doing so, bracing may negatively affect quality of life [8], as well as physical function over time [9].

Peak bone mass is attained in early adulthood, and about 90% of the adult bone mineral content (BMC) is deposited by the end of adolescence [10]. Late childhood and early adolescence are considered a critical period for attaining peak BMC [11], with nutritional intake and physical activity playing important roles in attaining peak BMC [12-14]. Adolescents with AIS are treated with braces during this critical period of bone mineralization [15]. This is of particular importance since girls with AIS have been shown to have low BMD compared to healthy girls of the same age and ethnicity [16-23].

To date, the few studies examining the effects of bracing on bone density [22,24-29] demonstrate inconsistent findings. For example, Snyder and his team found no differences in BMC between adolescent girls who had been braced and similarly-aged girls who were not braced [26]. Conversely, Courtois et al., reported lower bone mineral density in young adult women who had been treated with a brace during adolescence, compared with healthy similarly-aged women [29]. No comparison was made with women with AIS who had not been treated with a brace. Reasons for inconsistencies in previous studies include small sample size (e.g., n = 3) [22], short follow-up period from bracing to measurement of bone health [25,28], and no apparent control for, or account of physical activity and dietary intake [22,25-29]. In fact, of the studies that examined the effects of bracing on bone density, only three studies assessed physical activity. While the authors state that no differences in physical activity levels were observed between the AIS-braced and control groups, limited information on the methods used to assess physical activity and dietary intake are provided [25,26,29]. Since physical activity and nutrition play an important role in bone mass accrual during adolescence, it is important to account for their potential effects when assessing the effects of bracing on bone.

The purpose of the study was to assess the effects of brace treatment on BMC in adult women who were diagnosed with AIS and braced in early adolescence while accounting for current and past self-reported physical activity and nutritional intake. Additionally, previous studies examining the effect of bracing on BMC in adolescent girls or women, focused on the femur neck and lumbar spine. However, since physical activity may have localized effects [30], we also examined BMC in the upper and lower extremities. We hypothesized that BMC will be lower in young women with AIS who had been braced during adolescence and that this lower BMC will be more apparent in the lower limbs.

## Methods

### Participants

Using a non-experimental, cross-sectional design, we compared BMC in women who had been diagnosed with AIS and braced in their adolescence (AIS-B, n = 15) with that of women with AIS but did not receive any treatment (AIS, n = 15), as well as with that of healthy women (CON, n = 19). Participants were recruited through advertisements placed in bulletin boards, local newspapers and websites. All participants completed a medical screening (medical conditions, medications, family history of osteoporosis, fractures, extreme diets, age at menarche, previous pregnancies), and a scoliosis-related questionnaire (age of diagnosis, age of bracing, brace-wear duration). Inclusion criteria included Caucasian women aged 19–35 years at the time of measurement, as this period is characterized by relatively stable or slow declining bone mass. Exclusion criteria included current pregnancy, any disorder or medication intake known to affect bone mass or osteoporosis, and recent fractures. The study received clearance from the University’s Research Ethics Board and all participants signed an informed consent form.

### Measurements

Height was measured by a free standing stadiometer (SECA North America, USA), to the nearest 0.1 cm. Body mass was measured to the nearest 100 grams (EKS International Sweden AB, Sweden).

The 24-hour Nutritional Recall Questionnaire was administered as an interview to assess nutrient intake on a recent typical day. This questionnaire has been shown to provide valid estimates of total energy, calcium and vitamin D intake in adolescents and older adults [31,32]. Responses were analyzed by a single investigator, using Axxya System’s Nutritionist Pro Diet Analysis (Stafford, TX, USA).

Self-reported past physical activity was assessed using the long form of the Lifetime Physical Activity Questionnaire (LPAQ) [33]. This questionnaire provides information about the amount of occupation-related, household-related and exercise or sport activities that are of varying intensities from sitting to carrying heavy loads (see Table 1). Self-reported current physical activity (past 7 days) was measured using the Leisure Time Exercise Questionnaire (LETQ) [34]. This questionnaire provides information about the amount of vigorous, moderate or light activity, but does not provide details of specific activities. Previous studies have provided evidence of reliability and construct validity for scores derived from both the LPAQ [33] and the LTEQ [35].

**Table 1:** **Physical characteristics of the braced**, **not**-**braced and control group presented as means** ± **SD**

	**AIS** **-B ** **(** ***n*** ** = ** **15** **)**	**AIS ** **(** ***n*** ** = ** **15** **)**	**CON ** **(** ***n*** ** = ** **19** **)**	**ANOVA ** **(p-** **value)**
Age (yrs)	25.6 ± 5.8	24.0 ± 4.0	23.5 ± 3.8	.41
Height (cm)	167.3 ± 7.9	167.1 ± 7.2	167.3 ± 5.7	.99
Mass (kg)	63.1 ± 13.2	64.54 ± 10.2	65.2 ± 9.0	.85
BMI (kg/m^2^)	22.4 ± 3.3	23.09 ± 3.3	23.2 ± 2.6	.71
Body fat %	30.41 ± 6.8	30.8 ± 8.5	33.3 ± 7.6	.48
Total fat mass (g)	18748.0 ± 8234.0	19144.8 ± 8023.7	20810.2 ± 6892.5	.70
Total lean mass (g)	41089.0 ± 5722.3	41619.1 ± 5521.8	40565.2 ± 4396.1	.84
Age of menarche (yrs)	13.1 ± 1.7	13.0 ± 2.0	13.9 ± 1.4	.95

Note: Values are presented as means ± SD; there were no significant differences between groups (p > 0.05).

Body mass index (BMI) was calculated by dividing weight (kg) by height squared (m^2^).

All bone measurements were performed by the same technologist, using dual-energy X-ray absorptiometry (DXA, GE Lunar Prodigy). BMC was assessed at the spine, hip and the whole body. DXA was also used to assess body composition (percent body fat and lean mass). The hip and spine scans (Anterio-posterior) were obtained with the participant positioned supine on the densitometer table, with hips and knees flexed at 90°, to minimize lumbar lordosis. Whole body scans were obtained with the participants lying supine, with the legs internally rotated. Coefficient of variation for the femur neck, lumbar spine, and whole body BMC were 2.9%, 4.6 and 2.0%, respectively.

### Statistical analysis

The normality of the data distribution was confirmed according to the criteria by Tabachnick and Fidell [36]. Chi-square analysis was used to examine differences between groups in background medical information (e.g., past fractures, regularity of menses). Differences between groups in BMC, nutritional intake, physical activity and physical characteristics were examined using one way Analysis of Variance. The following variables have been shown through previous research to have an effect on bone parameters and were entered as covariates (ANCOVA) in examining group differences in bone characteristics: past and current physical activity [11-14,37-45], calcium and vitamin D intake [18,46-49] and lean body mass [50]. Pearson correlations were calculated to examine bivariate relationships between study variables. Statistical analyses were performed using SAS Ver. 16.0. Data are presented as means ± SD. Statistical significance was set at p < .05 (2-tailed).

## Results

Of the original 52 volunteers, three participants were excluded for not meeting age or ethnicity inclusion criteria. The final sample consisted of 49 Caucasian women; 19 CON, 15 AIS-B and 15 AIS. Participants in the AIS-B group reported brace treatment duration of 27.9 ± 21.6 months, for 18.0 ± 5.4 h/d. Participants in the AIS-B group reported using a Boston brace (n = 1), Milwaukee brace (n = 3), Charleston brace (n = 1), or a custom brace (n = 7). Three participants were unclear as to the type of brace used. Based on participants’ reports, it was assumed that all braces were rigid.

Chi-square analysis for personal and medical background data revealed no significant differences between the groups across any of the study variables. Chi-square values ranged from 0.25 to 4.73 (all p’s > .05). There were no significant differences in age, physical characteristics or age of menarche between the three groups (Table 2).

**Table 2:** **Daily Nutritional intake for the AIS braced** (**AIS**-**B**), **AIS not**-**braced** (**AIS**) **and control** (**CON**) **groups**

	**AIS-** **B** **(** ***n*** ** = ** **15** **)**	**AIS ** **(** ***n*** ** = ** **15** **)**	**CON ** **(** ***n*** ** = ** **19** **)**	**ANOVA (p-value)**
Total energy intake (kcal)	3218.9 ± 3952.3	2776.4 ± 873.3	1898.1 ± 667.6	.23
Calcium intake (mg)	1084.0 ± 637.8	1169.2 ± 863.4	1085.6 ± 621.9	.93
Dietary vitamin D (IU)	180.9 ± 145.9	194.2 ± 269.2	149.8 ± 149.6	.78

Note: Values are presented as means ± SD; there were no significant differences between groups (p > .05).

The mean reported Cobb angle of the two AIS groups at the time of bracing was similar (35 ± 11° vs. 38 ± 5° for AIS-B and AIS, respectively). However, we did not have this information for all participants (n = 8 for AIS-B and n = 13 for AIS). Therefore, at the time of testing we assessed the curve using a hand-held scoliometer. The Scoliometer angle which cannot be directly compared with the Cobb angle, did not differ between groups in the thoracic spine (10.3 ± 3.3 vs. 7.9 ± 6.0 for AIS-B and AIS, respectively), nor in the lumbar spine (5.4 ± 5.5 vs. 5.5 ± 6.1 for AIS-B and AIS, respectively). No correlation was observed between Cobb angle and femoral neck BMC, nor between scoliometer angle and BMC.

There were no significant differences between groups in daily total energy, calcium or dietary vitamin D intake (including supplements) (Table 3). All groups had mean calcium intakes above the recommended daily intake (DRI) of 1000 mg. However, 53% of the participants in each group had daily calcium intake below the DRI. Mean dietary vitamin D intakes were 25% to 32% of the RDI (600 IU).

**Table 3:** **Current and past physical activity for the AIS braced** (**AIS**-**B**), **AIS not**-**braced** (**AIS**) **and control** (**CON**) **groups**

		**AIS-** **B** **(** ***n*** ** = ** **15** **)**	**AIS** **(** ***n*** ** = ** **15** **)**	**CON** **(** ***n*** ** = ** **19** **)**	**ANOVA (p-value)**
Current physical activity (times/wk)	Mild	2.6 ± 1.7	3.2 ± 2.6	4.6 ± 3.6	.10
	Moderate	2.1 ± 2.0	3.0 ± 2.1	3.3 ± 2.5	.29
	Strenuous	2.0 ± 1.9	1.9 ± 1.9	2.9 ± 1.8	.23
Past physical activity (hrs/wk)	Intensity 1	0.1 ± 0.3	0.6 ± 1.5	0.1 ± 0.1	.18
	Intensity 2	1.1 ± 1.7	1.2 ± 3.1	1.0 ± 1.5	.96
	Intensity 3	1.5 ± 1.6	3.3 ± 2.7	2.9 ± 4.8	.33
	Intensity 4	3.8 ± 5.2	6.0 ± 5.9	5.3 ± 5.8	.57

Values are presented as means ± SD; there were no significant differences between groups (p > .05).

Mild = minimal effort (e.g., easy walking), moderate = not exhausting (e.g.,. fast walking), strenuous = heart beats rapidly (e.g.,. running, soccer, squash, basketball),

Intensity defined as:

1 = activities that require only sitting with minimal walking.

2 = activities that require a minimal amount of physical effort such as standing and slow walking with no increase in heart rate and no perspiration.

3 = activities that require carrying light loads (5–10 lb or 2–5 kg), continuous walking, mainly indoor activity and that would increase the heart rate slightly and cause light perspiration.

4 = activities that require carrying heavy loads (>10 lb or >5 kg), brisk walking, climbing, mainly outdoor activity, that increase the heart rate substantially and cause heavy sweating.

There were no significant differences between groups in the reported current and past physical activity (Table 1). Specifically, current physical activity showed no significant differences between the groups, although AIS-B tended to have engaged in less mild and moderate physical activity compared with AIS and CON groups, while both scoliosis groups tended to engage in less strenuous activity compared with CON. In terms of past physical activity, AIS-B had consistently lower physical activity at all intensities, but this difference was not statistically significant.

**Figure 1:**
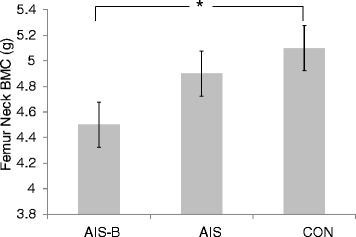
**Adjusted Femoral neck BMC after controlling for total lean body mass,** **calcium and vitamin D intake** **, past high intensity physical activity** **(Intensity 4)** **and current strenuous physical activity** **(mean ±** SD; * **p < .** **01).**

BMC and BMD data are reported from the dominant side. The pattern of results was similar for the two sides. None of the participants were considered osteoporotic in the femoral neck or in the spine. Four participants were osteopenic (T-score of −1.0 to −1.5) in the femoral neck region (two participants in the AIS-B group and one each in the AIS and control groups). Three different participants were osteopenic (T-scores of −1.5 and −1.6) in the spine (two participants in the AIS-B group and one in the AIS group). AIS-B had lower mean BMC and BMD in the lower extremities (Tables 4 and 5), although this difference was statistically significant only at the femoral neck axis (p = 0.03). Using ANCOVA, differences in mean BMC between groups were statistically significant (see also Figure 1) at the femoral neck and Ward’s triangle. No group differences were observed in mean BMC or BMD at the upper extremities or at the spine (all p’s > .05).

**Table 4:** **BMC values per skeletal site for AIS braced** (**AIS**-**B**), **AIS not**-**braced** (**AIS**) **and control** (**CON**) **groups**

	**AIS-** **B** **(** ***n*** ** = ** **15** **)**	**AIS ** **(** ***n*** ** = ** **15** **)**	**CON ** **(** ***n*** ** = ** **19** **)**	**ANOVA (p-value)**	ANCOVA* (p-value)
Arms	314.7 ± 74.6	324.4 ± 53.6	314.8 ± 33.2	0.85	0.84
Legs	911.4 ± 174.1	968.9 ± 195.2	963.7 ± 134.9	0.58	0.12
Pelvis	314.5 ± 75.7	347.4 ± 98.7	352.2 ± 64.6	0.36	0.16
Femur neck axis	2.1 ± 0.3^a^	2.3 ± 0.3	2.4 ± 0.34^a^	0.03	0.01
Femur neck	4.5 ± 0.1^a^	4.9 ± 0.6	5.1 ± 0.6^a^	0.06	0.02
Femur shaft	16.4 ± 2.0	16.9 ± 1.8	17.5 ± 2.0	0.24	0.05
Ward’s triangle	2.1 ± 0.5^a^	2.4 ± 0.5	2.5 ± 0.5^a^	0.11	.033
Spine-L1-L4	67.3 ± 13.0	67.5 ± 12.0	67.5 ± 10.2	0.99	0.55
Total body	2543.2 ± 522.5	2662.7 ± 502.1	2655.3 ± 323.8	0.71	0.27

^a^ = group effect.

* = Covariates included total body lean mass, calcium and vitamin. D intake, past strenuous physical activity (Intensity 4) and current strenuous physical activity.

Data are mean ± SD.

**Table 5:** **BMD values per skeletal site for AIS braced** (**AIS**-**B**), **AIS not**-**braced** (**AIS**) **and control** (**CON**) **groups**

	**AIS-** **B** **(** ***n*** ** = ** **15** **)**	**AIS ** **(** ***n*** ** = ** **15** **)**	**CON ** **(** ***n*** ** = ** **19** **)**	**ANOVA (p-value)**	ANCOVA* (p-value)
Arms	0.84 ± 0.12	0.85 ± 0.09	0.83 ± 0.04	0.83	0.58
Legs	1.21 ± 0.10	1.27 ± 0.11	1.26 ± 0.11	0.23	0.34
Pelvis	1.13 ± 0.11	1.14 ± 0.12	1.17 ± 0.09	0.56	0.78
Femur neck axis	0.92 ± 0.12^a^	0.96 ± 0.10	1.02 ± 0.13^a^	0.04	0.12
Femur neck	1.00 ± 0.11	1.02 ± 0.09	1.08 ± 0.10	0.07	0.17
Femur shaft	1.16 ± 0.12	1.20 ± 0.09	1.24 ± 0.14	0.24	0.24
Ward’s triangle	0.91 ± 0.12	0.93 ± 0.11	1.00 ± 0.15	0.08	0.16
Spine-L1-L4	1.17 ± 0.13	1.19 ± 0.09	1.22 ± 0.11	0.42	0.62
Total body	1.13 ± 0.09	1.15 ± 0.8	1.15 ± 0.06	0.67	0.84

^a^ = group effect.

* = Covariates included total body lean mass, calcium and vitamin. D intake, past strenuous physical activity (Intensity 4) and current strenuous physical activity.

Data are mean ± SD.

**Figure 2:**
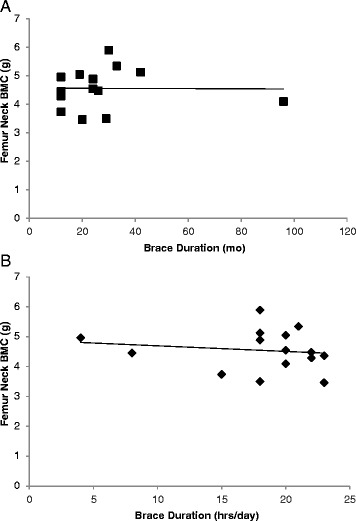
**Relationship between femoral neck BMC and reported duration of brace wear in total number of months (** **A,** **r =** **0.01,** **ns),** **and in hours/** **day (** **B,** **r =** **0.15,** **ns).** While most participants wore the brace for less than 3.5 years, one participant reported wearing the brace for 8 years (point on far right in top graph). Without this participant, the relationship between femoral neck BMC and reported brace wear in total months and in hours/day was r = 0.39 and r = −0.13, respectively (ns).

Pearson correlation coefficients between BMC at various skeletal sites and measures of physical activity, nutritional intake and lean body mass (LBM) are presented in Table 6. Overall, LBM was moderately-to-strongly correlated with BMC. Daily vitamin D intake was weakly correlated with BMC. There were no significant correlations between physical activity (current or past) and BMC. A similar pattern was observed between BMD at various skeletal sites and measures of physical activity, nutritional intake and LBM (data not shown). No significant associations were observed between BMD or BMC and reported brace wear in any of the skeletal sites (Figure 2).

**Table 6:** **Pearson correlations** (**r**) **between BMC and measures of physical activity**, **nutrition and lean body mass**

	**Strenuous physical activity**		**Nutritional parameters**			**Physical characteristics**
	**Current**	**Past**	**E I**	**Vit. D**	**Calcium**	**LBM**
Arms	0.17	0.03	−0.02	0.32*	0.23	0.71**
Legs	0.14	0.15	0.05	0.34*	0.25	0.72**
Pelvis	0.18	0.08	0.09	0.30*	0.12	0.56**
Femur neck axis	0.26	0.27	0.12	0.17	−0.07	0.46**
Femur neck	0.22	0.17	0.01	0.14	−0.03	0.53**
Femur shaft	0.26	0.22	0.13	0.30*	0.14	0.58**
Femur wards	0.13	0.16	−0.03	0.10	−0.20	0.50**
Total femur	0.23	0.18	0.10	0.30*	0.10	0.61**
Spine (L1-L4)	0.13	0.07	0.16	0.37*	0.14	0.68**
Total body	0.12	0.05	0.01	0.31*	0.20	0.60**

Note: *EI* = Energy intake, *Vit. D* = Vitamin D, *LBM* = Lean body mass.

**Correlation is significant at the 0.01 level (2-tailed).

*Correlation is significant at the 0.05 level (2-tailed).

## Discussion

This study assessed the effects of brace treatment on BMC in adult women who were diagnosed with AIS and braced in early adolescence. Our main finding was that after taking into account nutrition and physical activity, BMC was lower in the lower extremities in women with AIS who had been braced compared with healthy controls. However, no correlation was observed between BMC and reported brace wear duration, suggesting that other factors may be responsible for this lower BMC. It is suggested that one of these factors may involve weight-bearing physical activity. This study was novel insofar as potential confounding effects of physical activity and nutritional intake were taken into account, and BMC and BMD were evaluated at the upper and lower extremities, as well as the commonly assessed lumbar spine and femoral neck sites.

Femoral neck BMC was lower in the AIS-B compared to both, the AIS and the control group. This is in agreement with Courtois et al. [29] who examined the effects of bracing in a sample similar in age to our participants (age 30.5 ± 6 years), and reported that AIS-braced women had lower spinal BMD and consistently lower BMD at femoral sites than healthy women. No comparison was made to women with AIS who had not undergone brace treatment in the study reported by Courtois et al. [29]. The current study supports and extends Courtois et al’s [29]. findings by accounting for the plausible roles of dietary intake [18,46-49] and physical activity levels [11-14,37-45], which are known to effect bone accrual during adolescence.

Snyder et al. investigated the effects of bracing on BMD in adolescent girls, and concluded that brace treatment does not affect BMD at the spine and hip [25,26]. In the current study, group differences in BMD did not reach statistical significance except for the femur neck axis, which was lower in the AIS-braced groups. It should be noted that Snyder et al. [25] used a follow-up period of only one year and participants were still within their critical growth period. Additionally, while the overall rate of bone mineral accrual for the braced girls was not different than that reported for healthy adolescent girls of another study, the annual BMD increase at the femoral neck was lower in the braced girls [25]. Snyder et al. did not report rate of bone mineral accrual but their report of low BMD annual increase is in line with our findings of lower BMC and BMD in the femoral neck in the AIS-braced group. A similar pattern was observed in the femur shaft, Ward’s triangle and legs, although the differences between groups did not reach statistical significance.

In contrast to our hypothesis, we did not find any difference in the lumbar spine BMC or BMD between the three groups. While these findings are in agreement with previous studies [25,26], Courtois et al., observed lower BMD at the lumbar spine (L2-L4) in their AIS-braced group. The authors suggested that the low BMD was associated with the severity of the curvature of the spine [29]. In the presence of scoliosis, spinal BMD values, as determined by DXA in the traditional posterior-anterior direction, should be interpreted with caution because when the lateral curvature is accompanied by a rotation in the spine, as is the case in AIS, the curvature can affect the DXA results. DXA projects the three-dimensional bone structure into a two-dimensional image. Therefore, the measured BMD in the spine is likely to be negatively affected by any deformity or axial rotation of the vertebrae [51]. Girardi et al. demonstrated that the error can be as high as 20% [52]. Snyder et al. scanned six human vertebras in the sagittal plane and concluded that at axial rotations beyond 25 degrees, the pedicles came into view of the scan, influencing the bone parameters, and resulting in large errors in BMD and BMC values [25]. The differences between the frontal and sagittal plane spinal BMD ranged from 10 to 60% [25]. Cheng et al. [51] examined the effect of axial rotation of lumbar vertebrae on BMD and using DXA in the anteroposterior plane, with vertebral axial rotation in increments of 7.5 degrees, up to a maximum of 45 degrees [51]. Degree of rotation was negatively correlated with BMD, but not BMC. BMD decreased approximately 19% when the vertebrae were rotated by 45 degrees [51]. Those findings suggest that measurements of lumbar spine BMC are not affected by axial rotation, while BMD values may be underestimated. This suggestion is supported by findings that bone mineral measurements obtained from traditional posterior-anterior DXA were poorer predictors of vertebral ultimate load, compared with measurements obtained with lateral-projection DXA or micro-CT [53] Therefore, the lower spine BMD in the braced women, reported by Courtois et al., should be interpreted with caution.

Brace wear varied considerably among participants in terms of total duration, as well as the number of hours each day. For example, while most participants wore the brace for less than 3.5 years, one participant reported wearing the brace for 8 years. This may be regarded as a limitation to the study. However, it should be noted that despite the wide range, reported brace wear duration did not correlate with femur neck BMC. This lack of correlation was apparent even when excluding the participant with extended brace wear duration (Figure 2). Thus, it is suggested that other factors may be responsible for the lower femoral BMC observed in the AIS-B group. These factors may involve nutritional intake and type of physical activity.

Daily total energy intake, calcium intake and dietary vitamin D intake did not differ between groups nor did supplemental calcium and vitamin D intake. Moreover, no correlation was observed between calcium intake and BMC. This may be partly explained by the findings that most participants in this study reported sufficient calcium intakes and mean calcium intake in all groups was above the recommended daily intake. Plasma levels of 1,25(OH) vitamin D were not assessed. It is possible that plasma concentrations of vitamin D, rather than nutritional intake may have related to current bone mineral status. There were also no statistically significant group differences in levels of current and past physical activity although the AIS groups’ past and present physical activity tended to be lower, especially in the AIS-braced group. The questionnaires used in the present study, while reflecting general physical activity, unfortunately do not provide information specifically on weight-bearing high-impact activities (e.g., jumping, ball games). Therefore, we speculate that lower levels of *weight*-*bearing high*-*impact* activity specifically, rather than overall physical activity could partially explain the lower BMC in the lower-limbs of the AIS-B group, as recently suggested by others [54]. Their low BMC at the lower limbs and not at the upper limbs suggests that site-specific factors may be acting on the bone. The *set*-*point* for the effect of mechanical stress on bone, as defined in the Mechanostat theory [55], may not be constant and may vary from site to site [56,57], and between different activities [58]. Weight-bearing, high-impact physical activity is beneficial to bone accretion, especially at weight-bearing sites of the skeleton [37-45]. For example, Fehling et al. [59] demonstrated higher BMD in the legs and pelvis of gymnasts and volleyball collegiate female athletes compared with swimmers and non-athletes. Furthermore, following a 12 week jumping intervention program in children and adolescents, Johanssen et al. [60] reported a greater increase in leg BMC in jumpers vs. non-jumpers. We did not observe a significant difference in reported physical activity between groups. However, it is possible that brace wear does not adversely affect an individual’s ability to perform daily physical activity in general, but rather, it may hinder their ability to perform high-impact weight-bearing physical activity. Thus, we speculate that lower limbs’ bone accrual would be specifically affected. Indeed, Green et al. recently concluded that, although physicians encourage girls with AIS, with and without a brace, to participate in physical activity, the literature reporting such physical activity among girls treated with bracing is essentially anecdotal [61].

It should be noted that participants did not report the inclusion of specific scoliosis-related exercises in their treatment during brace wearing. Such physiotherapeutic scoliosis-specific exercises (PSSE) have recently been advocated for adolescents with idiopathic scoliosis [62]. The aim of these exercises is to reduce the lateral curvature of the spine and prevent curvature progression, but it does not appear that these exercises are aimed at preventing bone mineral loss or enhancement of bone mineral accrual. Thus, it is suggested that treatment for AIS, and especially brace treatment, should include PSSE, as well as weight-bearing physical activity that may prevent bone mineral loss and promote bone mineral accrual [54].

There are several limitations to this study. Since this is a cross sectional study we are unable to make causal inferences with confidence. Thus, we cannot rule out the possibility that the AIS-B group had low BMC in the lower limbs before diagnosis and brace treatment. However, the fact that no group differences were observed in BMC in the upper limbs suggests that the lower BMC in the lower-limbs may be related to the brace wearing. Additionally, there are important potentially confounding factors, such as age of menarche, endocrine disorders, extreme diets, history of osteoporosis, on which information was collected retrospectively. While these data were taken into account in participant inclusion or in the comparison between groups, a prospective study would be needed to examine the influence of these factors on the effects of bracing. The relatively small sample size and low statistical power may have affected the strength and associations of our findings and the probability of finding a statistically significant difference between groups. Nevertheless, despite the relatively small sample size, group differences in BMC were observed in the femoral neck axis, with a similar pattern in other skeletal sites. This overall pattern suggests that brace wear may affect bone accrual during adolescence indirectly, possibly through its effect on the pattern of physical activity. Finally, past dietary intake was not assessed in the current study. While such data could have been insightful, it should be noted that recall of food intake over a long time period is very difficult.

## Conclusion

Young women with AIS, especially those who were treated with a brace during their growing years, have significantly lower BMC in the lower limbs compared to women without AIS. However, the lack of a relationship between bracing duration during adolescence and BMC during young adulthood suggests that the brace treatment is not the likely cause of the low BMC.
